# The impact of the COVID-19 pandemic on nursing students’ navigation of their nursing programmes and experiences of resilience. A qualitative study

**DOI:** 10.1016/j.ijnsa.2023.100146

**Published:** 2023-07-28

**Authors:** Catherine Henshall, Zoe Davey, Clair Merriman, Laura Strumidlo, Laura Serrant, Jo Brett, Eila Watson, Jane V. Appleton, Mary Malone, COV-ED Nurse Study Group, Carrie Bradbury-Jones, Sarah Bekaert, Dan Butcher, Paul Dawson, Daniel Kelly, Sonja Mcllfatrick, Kinga Papiez, Anne Marie Rafferty, Pras Ramluggun, Mike Ramsay, Lynn Sayer, Marion Waite, Tessa Watts, Cate Wood

**Affiliations:** a University of Birmingham, UK; b Oxford Brookes University, UK; c University of Cardiff, UK; d University of Ulster, UK; e Kings College London, UK; f University of Dundee, UK; a Oxford Institute of Applied Health Research, Oxford Brookes University, Oxford, UK; b Research and Development, Oxford Health NHS Foundation Trust, Oxford, UK; c University of Coventry, Coventry, UK; d Health Education England (North East & Yorkshire), Sheffield, UK; e Kings College London, London, UK

**Keywords:** Nursing, Resilience, Nurse education, Training, Support networks, Qualitative, Students

## Abstract

**Introduction:**

High-quality pre-registration student nurse training and development is integral to developing a sustainable and competent global nursing workforce. Internationally, student nurse recruitment rates have increased since the onset of the COVID-19 pandemic; however, attrition rates for student nurses are high. During the pandemic, many student nurses considered leaving the programme due to academic concerns, feeling overwhelmed, and doubting their clinical skills. Little was known about the extent to which nursing education prior to COVID-19 had prepared students for their role in managing the healthcare crisis or the impact on their resilience. Thus, this study aimed to explore how the COVID-19 pandemic impacted on the resilience levels of student nurses across the United Kingdom.

**Methods:**

Data were collected as part of a multi-site qualitative study named ‘COV-ED Nurse’ and involved pre-placement surveys, placement diaries, and post-placement interviews with nursing students. Student nurse participants were recruited from across the United Kingdom, from all years of study, and from all four nursing branches: children, adult, mental health, and learning disabilities. Participants were asked to complete a pre-placement survey that collected demographic details and information about their placement expectations. They were also asked to record a weekly audio-visual or written diary to describe their placement experiences, and, on completion of their placements, students were interviewed to explore their experiences of this time. Data were thematically analysed using the Framework Approach. Ethical approvals were obtained.

**Results:**

Two hundred and sixteen students took part in the wider study. The current study involved a subset of 59 students’ data. Four main themes were identified: ‘coping with increased levels of acuity’, ‘perceived risks of the pandemic’, ‘resilience when facing uncertainty and isolation’, and ‘the importance of coping mechanisms and support structures.’

**Discussion:**

From this study, we have generated insights that can be applied to nursing research, education, policy, and practice and identified the wide-ranging impact that the COVID-19 pandemic had on student nurses and their abilities to remain resilient in an unstable environment. The value of communication and support networks from a wide range of sources was highlighted as key to navigating many uncertainties. In addition, the extent to which students were able to navigate their personal and professional roles and identities influenced their ability to cope with and continue along their training pathways.

## 1 Contribution of the paper

**What is already known about this topic?**

• High-quality pre-registration student nurse education is integral to the development of a sustainable and competent global nursing workforce.
• During the COVID-19 pandemic, many student nurses considered leaving the programme due to academic concerns, feeling overwhelmed, and doubting their clinical skills; however, little is known about the impact of the pandemic on their resilience.

**What this paper adds**

• COVID-19 greatly impacted nursing students and their resilience; however, good communication and support networks were key to navigating personal and professional roles and identities.
• Higher education institutions should maintain strong links with healthcare organisations so that students can take advantage of robust communication mechanisms, mentorship and supervision, and flexible learning environments.

## 2 Introduction

High-quality pre-registration nurse education is integral to the development of a competent, sustainable, and robust nursing workforce (Aiken et al., 2014). Internationally, student nurse recruitment rates have increased since the COVID-19 pandemic onset (although at a smaller percentage increase than in previous years), perhaps due to the heightened media profile of nurses, which has exposed the highly accomplished and comprehensive skill sets that they possess (Bagnasco et al., 2020). In the United Kingdom (UK) in 2021, total applications for nursing courses rose by 32% (*N* = 60,130) compared to the previous year, with 20,930 students successfully gaining a place on a nursing degree programme (nurses.co.uk, 2021; Universities and Colleges Admissions Service, 2021). This trend has been seen internationally, including in the United States of America (USA), where nurse student enrolment in entry-level baccalaureate programmes increased by 3.3% in 2021, compared to the previous year (American Association of Colleges of Nursing, 2022). Despite this growth in applications, an increased number of prospective students have delayed the start of their education programmes (deferral), alongside a rise in attrition rates and numbers of nursing students taking a break in their education (Jones-Berry, 2021). This has resulted in a reduction in qualified nurses entering the workforce, with one in three nursing students in the UK failing to complete their degree programmes within the 3-year timescale (Jones-Berry, 2021); in Australia, the reported yearly attrition rate was 17% (Wilson, 2022). There is concern that increases in recruitment to nursing programmes may not be sustainable due to global realities around the cost-of-living crisis and industrial action in the UK, with unrest around nurses’ pay and working conditions deterrents to uptake (Mahase, 2022). To address this workforce crisis and improve nursing retention rates, it is important to understand which factors contribute to the development and maintenance of resilience in nursing students to help support and retain them as they progress through their careers.

Resilience can be defined in many ways and is a fluid construct, changing over time, depending on the internal and external stressors that individuals are exposed to (Davey et al., 2020; Jackson et al., 2007). A common definition of resilience that is specific to student nurses states:

“Nursing student resilience is an individualised process of development that occurs through the use of personal protective factors to successfully navigate perceived stress and adversities. Cumulative successes lead to enhanced coping/adaptive abilities and well-being.” (Stephens, 2013, p130).

**Fig. 1:**
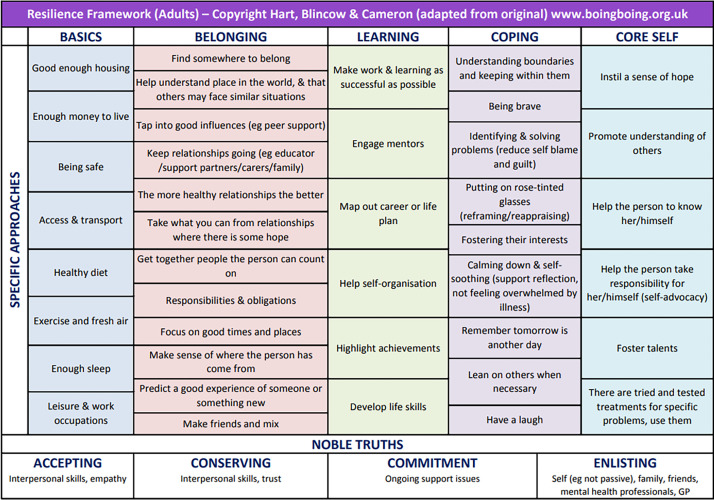
Hart et al. (2012) Resilience framework.

The Hart et al. (2012) Resilience Framework (Fig. 1) goes further by outlining different components in an individual's life that influence and inform the state of resilience. Basic needs, such as housing, finance, safety, healthy food, fresh air, sleep, and work/life balance, are essential components and underpin other factors, such as self-care and well-being (Hart et al., 2008). However, despite the Resilience Framework, there is no consensus on the precise meaning of the term ‘resilience’, not only within and between nurse education and nursing but also in other populations (Hart et al., 2016), and it is apparent within the resilience literature that multiple theories, models, and definitions of resilience are used and frequently debated (Wiig et al., 2020). There is also criticism of the requirement for individual resilience to compensate for deficits in organisational resources, such as safe staffing levels and working conditions (Traynor, 2017).

The capacity to locate and navigate resources to overcome adversity and to thrive is an essential part of resilience development and is central to nurses’ ability to practise safely and effectively within stressful and high-pressured environments (Hart et al., 2016; NMC, 2018, a). The term ‘resilience’ covers physiological, psychological, and sociocultural aspects of the self, with some people having more widely developed strategies for enhancing their resilience and protecting their mental and physical health (McDonald et al., 2012). The way resilience is experienced, modified, and utilised has implications for how the future nursing workforce can progress and be sustained under increasingly complex working environments. As this study explored the experiences of individuals, it was appropriate to focus on this resilience concept from the viewpoint of participants. However, it is recognised that resilience formation is widely influenced by other external, context-specific factors, such as being a nursing student during the pandemic, as well as by the institutional support provided by academic and clinical institutions. Knowledge regarding resilience formation is also valuable given its increasing prominence within recent nurse education and nursing standards (NMC, 2018b). Yet there have been few studies in this area, with a paucity focusing explicitly on student nurse resilience (McGowan and Murray, 2016). Those that have been conducted were pre-pandemic, and none described the process of becoming resilient.

Nurse education in the UK is regulated by the Nursing and Midwifery Council, which follows the European Directive 2005/36/EC (World Health Organisation, 2009). This requires that nurse pre-registration programmes must total 4600 h. Of these, 2300 must be practice hours, achieved through clinical placements. These are challenging to source due to increasing nursing and multi-professional student numbers and a heavy workload for those supporting students. The requirement for both university modules and clinical placements is a common reason for attrition, with exposure to challenging placement experiences, poor attitudes of placement staff, and a lack of support from clinical supervisors, assessors, and teams being identified as factors influencing programme withdrawal (Eick et al., 2012; ten Hoeve et al., 2017). In England, a national study on attrition in healthcare students, including nurses, found that failure at assessment, wrong career choice, and financial reasons were leading causes of attrition (Health Education England, 2018). During the first wave of the pandemic, nursing students (37%) considered leaving the programme due to academic concerns, feeling overwhelmed, and doubting their clinical skills (Health Education England, 2020). This figure was higher than for allied health professional students (27%), but not so high as for midwifery students (41%). More evidence is required to understand the best ways to build and bolster nursing students’ resilience and minimise attrition by developing strategies for navigating resources that can enable them to thrive.

Challenges relating to nursing students’ educational experiences and subsequent attrition rates have been compounded during the COVID-19 pandemic, set against the backdrop of a nursing workforce crisis. Internationally, the pandemic led to unprecedented health and care system challenges with significant implications for the nursing workforce (Charles et al.,  2021; Shembavnekar,  2021). Frontline healthcare staff worked under pressurised conditions to deliver expert patient care in an environment of increasing staff sickness and absence and rapidly changing infection control measures and policies, as well as increased patient acuity (Deogon et al., 2020) and new and evolving patient care complexities (Vindrola-Padros et al., 2020; Willan et al., 2020). The pandemic involved immediate changes to nurses’ working practices through social distancing measures, redeployment, personal protective equipment, and constantly changing guidance and protocols aimed at minimising the spread of the virus (Zhou et al., 2021). Many of these measures, to a greater or lesser extent, have remained in place globally across health services since 2020; rapid and stressful changes to nursing care delivery have challenged the resilience and psychological well-being of the workforce, with increased levels of stress, distress, anxiety, and depression reported (Morgantini et al., 2020; Courazze et al., 2021; Ferry et al., 2021). The pressures faced by qualified nursing staff are likely to impact nursing students who are working side-by-side with exhausted workers, which may harm their own resilience, professional outlook, and skills development.

With increased demand for nursing care during the pandemic, the Nursing and Midwifery Council recognised nursing students’ potential contribution by issuing emergency educational standards (NMC, 2020). The standards permitted nursing students in the final two years of their programme entry to the nursing workforce as paid members, losing supernumerary status (which allowed students to be present in excess of the normal or requisite number of qualified nurses), but retaining protected learning time, appropriate supervision, and support (NMC, 2020). Little was known about how nurse education prior to COVID-19 had prepared nursing students for their rapidly evolving role in managing the healthcare crisis or the impact it would have on their psychological health, well-being, and resilience. We report findings not only from a large-scale qualitative study that explored how nurse education prepared UK nursing students prior to and during COVID-19 but also from a sub-study to explore how the pandemic impacted nursing students’ resilience and the process of their becoming resilient.

## 3 Methods

### 3.1 Design

Data for the sub-study were collected as part of a UK-wide multisite qualitative study named ‘COV-ED Nurse’ (https://www.brookes.ac.uk/research/units/hls/projects/cov-ed-nurse/). It consisted of a longitudinal qualitative study with three consecutive phases: pre-placement surveys, placement diaries, and post-placement interviews with nursing students.

### 3.2 Recruitment

Recruitment took place between November 2020 and July 2021. Student nurse participants were recruited from the UK and from all four nursing specialties: adult, children, learning disabilities, and mental health (including those who were dual-trained). A hub-and-spoke approach was used to aid recruitment, coordinated via a centrally managed study website. The study was then promoted via existing networks, which included project partners and steering group members across the UK. Eligible nursing students from the four UK nations were targeted using a variety of methods, including social media networks, university lecturers and student unions, online learning platforms, and word of mouth. Convenience and snowball sampling techniques were used. All nursing students who were due to start a clinical placement in any year of their degree programme were eligible to participate. Participants were encouraged to promote the study to their student nurse peers to aid recruitment. Eligible students were invited to complete an online expression of interest form and were provided with a link to the study website where they could access a participant information sheet. Interested students then submitted their online expression of interest form and were provided with an online consent form to complete. To incentivise recruitment, all participants who completed the survey and diary phase of the study were given a £20 voucher, and participants who completed all phases of the study were entered into a prize drawing to win an iPad.

### 3.3 Data collection and analysis

Data collection took place between December 2020 and August 2021 and consisted of the following phases.

#### 3.3.1 Phase 1: pre-placement survey

Participants were asked to complete a short online pre-placement survey (Appendix 1). The survey collected information related to participants’ demographics, upcoming clinical placement area, expectations of the placement, and how well-prepared they felt. Survey data were analysed using IBM SPSS Statistics for Windows, Version 27 (Armonk, NY: IBM Corp.). Descriptive statistics were used to summarise the data and to provide context for themes emerging from the qualitative data.

#### 3.3.2 Phase 2: placement diaries and post placement interviews

Participants were asked to record a weekly audio-visual or written diary over four consecutive weeks of their clinical placement to describe their experiences. Diaries were recorded or written using participants’ own devices (e.g., smartphones, tablets, laptops) and were sent, via weekly uploads, to the central team via a Google form. Participants were provided with instructions on how to set up a Google email account and make and upload recordings. They were also provided with a broad topic guide, which asked them to describe their experiences and reflect on how they felt their university and clinical nursing education prior to and during COVID-19 had influenced their resilience and coping abilities, both personally and professionally.

On completion of their clinical placements, students were contacted by the central team to take part in a one-hour, post-placement, one-to-one interview, which was conducted online using the Zoom platform. Five members of the research team (CW, PD, KP, CM, DB), who were not known to participants, conducted the interviews; all held doctoral qualifications and were nurses or healthcare researchers interested in the topic. They used a topic guide, informed by the placement diaries, to discuss students’ experiences and reflections. The interviews explored the impact of working through COVID-19 from a professional and personal perspective, the perceived support that was offered to students, and how their experiences influenced their decision-making relating to a future career in nursing.

Weekly audio-video recordings or written diary records and interview data were transcribed by a transcription company, and an inductive thematic analysis was undertaken using the Framework Approach (Gale et al., 2013). Participant data (either interview or diary data) were analysed together as one overarching dataset within a framework matrix. NVivo software version 12 was used to manage the data. Double coding was undertaken on a selection of transcripts (ZD, JB, PD, CW, KP) to ensure rigour and consistency. Weekly research team meetings were held to develop the coding tree, ensure analytical rigour, and agree on interpretations of the dataset. Ethical approvals were obtained in May 2020 from the central research team's local university research ethics committee (L20209). All participants provided online written informed consent prior to participating in the study, as well as recorded verbal consent prior to taking part in the interview phase. All surveys, diary, and interview data were de-identified prior to analysis and stored in secure, password-protected Google Drives. All data were managed according to General Data Protection Regulation principles.

## 4 Results

In total, 216 students consented to take part in the main study; of these, 124 took part by submitting one to four diaries, taking part in a post-placement interview, or both. The sub-study reported on in this paper involved the analysis of a subsample of data contained within three relevant sections of the inductive framework matrix related to the concept of resilience (emotional impact, support, and coping strategies). The subsample comprised data from 59 participants, including 51 diary entries and 45 interviews. Demographics of these 59 participants are presented in Table 1. Most participants were White; this is disproportionate to the numbers of non-White students across the nursing workforce and is important to recognise given the additional health concerns for healthcare workers from non-White backgrounds during the pandemic. It is possible that a more proportionate representation may have resulted in different findings. The median age of participants was 23.5 years (Interquartile Range (IQR): 20.25–34.00).

**Table 1:** Participant demographics (*N* = 59).

			n (%)	
Region	England (South)			9 (15.3)
	England (Midlands)			10 (16.9)
	England (North)			7 (11.9)
	England (London)			16 (27.1)
	Scotland			6 (10.2)
	Wales			8 (13.6)
	Northern Ireland			3 (5.1)
Nursing Field	Adult Nursing			40 (67.8)
	Children's Nursing			9 (15.3)
	Mental Health Nursing			6 (10.2)
	Learning Disability Nursing			2 (3.4)
	Dual Field (mental health/adult; child/mental health)			2 (3.4)
Year of Study	Year 1			3 (5.1)
	Year 2			25 (42.4)
	Year 3			31 (52.5)
Gender	Female			55 (93.2)
	Male			3 (5.1)
	Non-binary			1 (1.7)
Ethnicity	White			50 (84.7)
	Black			3 (5.1)
	Asian			4 (6.8)
	Chinese			1 (1.7)
	Other			1 (1.7)
Relationship Status^1^	Single			23 (40.4)
	In a relationship			20 (35.1)
	Married/defacto/civil partnership			12 (21.1)
	Divorced/separated			2 (3.5)
Children^1^	Yes			16 (28.1)
	No			41 (71.9)
Other dependents^2^	Yes			4 (7.3)
	No			51 (92.7)
Feelings ahead of placement	Excited			34 (57.6)
	Apprehensive			25 (42.4)
	Motivated			18 (30.5)
	Nervous			36 (61.0)
	Frightened			8 (13.6)
	Determined			24 (40.7)
Feeling of preparedness^2^	Very well prepared			2 (3.64)
	Well prepared			18 (34.6)
	Somewhat prepared			27 (47.3)
	Not very well prepared			6 (10.9)
	Not at all prepared			2 (3.64)

1. *n* = 57 (Missing/not stated *n* = 2) 2. *n* = 55 (Missing/not stated *n* = 4).

## 5 Summary of findings

Four main themes were identified. The first three related to nursing students’ experiences of working clinically during the pandemic and the impact on their resilience. These were ‘coping with increased levels of acuity’, ‘perceived risks of the pandemic’, and ‘resilience when facing uncertainty and isolation’. A fourth major theme, ‘the importance of coping mechanisms and support structures’, related to the variety of coping mechanisms participants utilised through internal, external, and self-managed support networks, which drew on peers, friends, and family. Each theme is explored in more detail below.

### 5.1 Theme 1: coping with increased levels of acuity

Approximately one third of nursing student participants reported that one of their biggest challenges related to increased levels of patient acuity, with many faced with an influx of sick COVID-19 patients who were rapidly declining in health or experiencing sudden death. The volume of patients who were experiencing sickness, ill health, and death due to the virus led to a perceived normalisation of death from COVID-19, which many participants found hard to deal with.

*“This volume of deaths, like it being so kind of almost normalised, that's the kind of thing I struggled with. I think because everybody had been dealing with at least…I think it was like at one point a death every day almost for them…I think it was just a self-preservation technique for the staff, that it was just another day. Whereas I kind of struggled with that aspect.” Participant S3 (Third Year Adult Nursing Student, Scotland)*

Over a third of participants spoke about the enormity of the situation and the expectations placed on them to deal with the new pressures they were under. However, these pressures were recognised as being integral to their resilience and development as nurses.

*“It just felt like I was a rock dropped into the ocean, and I was expected to come up swimming…We are going to be like the Swiss army knives of nursing because we will have coped with so much, we are going to be your resilient nurses, we are going to be your nurses that are ready for anything” Participant ES72 (Second Year Adult Nursing Student, England South)*

The isolated nature of the process of patients dying was reported to test participants most; this was because often patients were not allowed visitors, so their final moments were then spent with clinicians, including nursing students. This had a powerful effect on students who employed various coping strategies to sustain their resilience in these circumstances.

*“I've seen some things I don't think I'm ever going to forget, and I have to carry it all with me going forward…So, whether I can cope with it day-in and day-out, I'm not sure…I try and keep a diary at home of how I feel throughout the week and anything I would like to reflect upon, I feel as if this helps me cope. I also have a strong family unit who are supportive, and I can talk to them about any anxiety as well.” Participant W1 (Second Year Adult Nursing Student, Wales).*

However, over a third of participants were able to positively reframe these challenging experiences by finding strength and learning from the situation to move forward. They commented that having to cope with difficult situations reaffirmed the need to draw on their own reserves of resilience to progress as a nurse. They also expressed admiration for the nurses they were working alongside, whom they often role-modelled.

*“Oh, it's just amazing what the nurses do. You have to be so strong to do that job, it's such an amazing job. Just like you're constantly busy in the acute wards, you've got so much to do.” Participant S9 (Second Year Mental Health Nursing Student, Scotland)*

### 5.2 Theme 2: perceived risks of the pandemic

In their roles as nursing students, many participants identified new risks resulting from the pandemic. These included risks to their health, with some students describing concerns relating to receiving the COVID-19 vaccine, as well as concerns about infection control measures. This caused increased stress that was dealt with individually, with or without employing internal or external resources.

*“Thinking about COVID-19, I am still not happy to take the vaccine as I feel it might have an effect on me later…And I keep testing twice a week to make sure I don't spread any infection to vulnerable children in the ward and people around me.” Participant EL102 (Second Year Children's Nursing Student, England London).*

Participants also expressed fear for their qualified nursing colleagues due to the increased risk that nurses were under at the start of the pandemic, caring for patients without sufficient protection from the virus; this had led to some colleagues losing their lives.

*“Because obviously at the very start, and all these amazing nurses were dying going to work, that really upset me.” Participant EN70 (Third Year Adult Nursing Student, England North)*

Nursing student participants expressed anxiety that they were risking the lives of patients they were caring for due to the increased risk of virus transmission from contacts in day-to-day life. In addition, participants voiced concerns that by working in their roles they were potentially placing families and friends at risk.

*“Not so much from them giving me anything, but I was a lot more concerned about passing on something to them, because obviously they're more vulnerable. I was quite aware of like even when I wasn't on placement, you know, what was I doing at home, what was I going to be bringing into these people.” Participant NI3 (First Year Mental Health Nursing Student, Northern Ireland)*

### 5.3 Theme 3: resilience when facing uncertainty and isolation

Varying uncertainty experienced by participants was often associated with changes that had occurred since the onset of the pandemic. Many felt the perceived loss of a normal nurse education, having never or only partially experienced this. This included a suspension of face-to-face teaching, which led to feelings of isolation, whilst others struggled with the introduction of paid placements. Changes to the way that nursing courses were delivered, with increases in remote learning, led to a loss of engagement and social contact with peers and tutors.

*“It was like eight, nine hours a day of recorded lectures. Yeah, so it was quite, it was quite overwhelming…It was absolutely gruelling” Participant W19 (Second Year Adult Nursing Student, Wales)*

Constantly changing guidance and delays in decision-making relating to nursing student courses and placement areas also introduced uncertainty for many participants.

*“That was the scariest time because when everything was happening, the universities wouldn't make a decision, the government wouldn't make a decision.” Participant EN24 (Second Year Adult Nursing Student, England North)*

Several participants commented that they felt proud that they had continued and contributed to a moment in history and made a visible contribution at a time of national need, which appeared to bolster their resilience.

*“[I] felt like it was nice to say to myself that I'd done this to help out in this huge pandemic that the world is going through, and in years to come I'll be able to say when I was a student, I decided to opt in and work and do my bit to help out in the pandemic, so that was a nice feeling.” Participant EN107 (Third Year Adult Nursing Student, England North)*

Others reported experiencing internal conflict regarding continuing their clinical placements during the pandemic, with mixed emotions at play. One participant described how this impacted their resilience and their overall health.

*“I feel like that [resilience] wasn't at the same level as the pandemic went on, and especially with kind of the time constraints of when I was on placement, combined with winter, combined with being really busy, I felt like that just kind of reached a point where I was like yeah, I probably should see a professional about this now.” Participant EN3 (Second Year Mental Health Nursing Student, England North)*

The success or failure of relationships participants had with staff in placement settings was often reported to be contingent on the attitude and actions of the staff members involved. These relationships were tested by the pandemic, with issues around staffing, social distancing, and COVID-19 policies, as well as changes to usual practice and procedures, all impacting the perceived quality of support provided. Students understood the additional pressures that staff were under but still expected a certain degree of personalised contact between themselves and their academic tutors and clinical supervisors/assessors; this was sometimes absent and could result in unsatisfactory experiences for students.

*‘I feel as though on some shifts I felt very supported by some staff, but, on other shifts, my supervisor would not support me emotionally or practically. On these days I felt very forgotten about which was very frustrating because I felt like I was missing out on learning opportunities and had no guidance’ Participant EM12 (Second Year Adult Nursing Student, England Midlands)*

### 5.4 Theme 4: the importance of coping mechanisms and support structures

Participants reported that their support needs in clinical placements during COVID-19 were high, and the impact on their personal resilience was considerable. Support structures were crucial to students’ ability to cope with the competing stressors of being on placement, managing their academic coursework, and the pressures raised by the pandemic. Students identified internal and external support structures that provided necessary scaffolding for navigating new and evolving clinical and academic experiences. They also reported undertaking self-managed activities to aid their resilience and help them recharge.

### 5.5 Internal support structures

When supportive relationships developed with clinical staff, supervisors, and assessors, they were reported to be valuable in helping students feel part of a workplace community, connected to the nursing profession, and supported whilst on placement. This development of open and trusted relationships characterised this structured support.

*“I think having [a clinical staff member] there made a huge difference because one, I kind of felt part of her team…We were part of a team. And that was like, she gave me support, gave me a sense of belonging, sense of community, but…It reminded me that actually I do want to be a nurse.” Participant S3 (Third Year Adult Nursing Student, Scotland)*

Participants also discussed structured support in academia, which included relationships with university tutors and course coordinators. The purpose of these relationships was to manage students’ expectations and aid their preparedness, to debrief around specific incidents, and to provide learning support.

*“I did email my academic adviser…And they were really good, if you had any questions about deployment, even though a lot of the time they don't know the answers; actually if you got in touch with them, they were always really helpful, and they gave us as much information as they could as soon as they got it…It was just a bit of a chaotic situation, nobody knew quite what was going on yet.” Participant EN16 (Third Year Adult Nursing Student, England North)*

Participants commented on the availability of formal support sessions in clinical and academic settings. Whilst they were aware of this provision, they did not often report accessing the sessions, stating they were unnecessary. The knowledge that formal support was available was reported to be reassuring to some; however, others found formal sessions often onerous, inconsistent, impersonal, and unhelpful.

*“The Education team at the trust has started to do these extra check-in sessions, which have turned out to be more of a burden than a support because it's just going through the same questions for people. They're mandatory to attend once a week. They're not the environment to be, for me, personally, sharing my feelings, reflections, because it's a lot of people on a Zoom call.” Participant EL42 (Third Year Adult Nursing Student, England London)*

### 5.6 External support structures

External support included that sought by participants outside their nursing courses. This could include formal support, such as seeking help from mental health professionals, and informal support provided by friends, family, and peers. The most reported coping strategy was talking to other people about shared experiences. Participants generally had a diverse network of people they could share their experiences with, and common modes of communication included face-to-face (where restrictions allowed) and virtual modes, such as Zoom, Facetime, telephone, and social media. Participants indicated that family and friends were highly valued sources of support, providing comfort, validation, and distraction from the difficult pandemic experiences presented to them.

*“I have a lot of support from my boyfriend, my mum, my family, and my friends, and a lot of people close to me are kind of really, you know, like complementary of what I'm doing, and will often say, you know, that's amazing…So, I do feel supported.” Participant EL128 (First Year Adult Nursing Student, England London)*

However, participants also noted that sometimes family and friends were unable to fully understand the nature of these experiences or how to respond to them. For this reason, participants often sought support from family and friends who were involved in healthcare to draw on shared understandings and make sense of common experiences.

*“So those friends are friends that I made right at the beginning of my course, and we obviously got close, and we are living together now. And I think we've just got stronger throughout, especially…choosing to go on placement. When we were in that period, there were just three of us in the house by ourselves, and obviously we were in lockdown, we couldn't really go out anywhere. We were just going to placement and then coming home, and we definitely got really close” Participant W31 (Third Year Adult Nursing Student, Wales)*

Participants also discussed the value of peer support within the clinical context, emphasising the importance of shared experiences and understanding between peers within the same cohort.

*“My classmates…They've been the biggest support throughout this whole thing, this whole two-year training, and I say that they continue to be this time.” Participant EL42 (Third Year Adult Nursing Student, England London)*

The pandemic substantially impacted peer-to-peer relationships due to the cessation of face-to-face lectures and meetings and changes in student numbers in placement settings. This led to participants identifying a perceived gap at times, as they recognised that the peer support they relied on was not always as readily available as pre-pandemic.

*“It has been difficult not being able to see my student nurse friends to talk about placements during COVID-19. It's such a crucial part of student nurses coping, as no one else really understands your experiences. I have many people in my family that work in healthcare, but it's still better to talk to student nurses, and it isn't the same doing this virtually.” Participant EL49 (Third Year Adult Nursing Student, England London)*

Very few participants reported seeking external professional support, but on occasion this was reported as being necessary.

*“I had taken a few days off placement during the first week for my mental health and well-being…I had referred myself to therapy…I was not aware, prior to starting this placement, I was going to be working with children who were previously exposed to COVID-19. It also made me feel anxious considering I live with my parents and one of my siblings” Participant EL67 (Third Year Children's Nursing Student, England London)*

### 5.7 Self-management support structures

Many participants reported self-management strategies they had utilised to help them cope with the pressures they were experiencing. They reported that planning and undertaking activities helped them cope better when they returned to their placement areas. Due to the restrictions imposed by the pandemic, these activities were often limited. However, where possible, they included meeting up with friends and family and often included distraction therapies or techniques to help them unwind after a busy placement. The most common distraction technique was exercise or physical activity, with walking, running, or biking commonly cited, as well as meditation, mindfulness, yoga, listening to hypnotherapy tapes, drawing, listening to music, reading, cooking, and watching television. All were reported as helping participants to refocus and distance themselves from their often gruelling, clinical experiences.

*“I practise a lot of meditation and mindfulness, which helps me stay calm during my shifts” Participant W19 (Second Year Adult Nursing Student, Wales)*

Participants also reported accessing a variety of online apps that had been recommended by colleagues. One participant reported that their university had hosted a webinar run by a healthcare professional that provided mental health information and advice for students.

*“He gave us some methods, like the pivot method and grounding. I've used them and they really helped” Participant EM139 (Second Year Adult Nursing Student, England Midlands)*

Some participants reported how writing either in a journal or writing reflective pieces (both independently and as a study requirement) reduced their anxieties by helping them absorb and process their placement experiences. These participants commented that when writing, they tried not to focus purely on negative experiences but also on things that had gone well. Participants talked about how they constructively reflected each day on something they had achieved, enabling them to maintain a level of positivity and resilience.

*“Writing reflection of this placement this week, and I feel this will help me process my thoughts about the whole thing” Participant W1 (Second Year Adult Nursing Student, Scotland)*

“*I did think about something that had gone well, and I did think about something that was hard. I think acknowledging that actually is really, really helpful” Participant EL128 (First Year Adult Nursing Student, England London)*

## 6 Discussion

The techniques for coping and support utilised by study participants resonated with the Hart et al. (2012) Resilience Framework (Fig. 1). The Resilience Framework has been used to work with people to build personal resilience by focusing on a positive, or asset-based, approach, playing to people's strengths, and encouraging areas for development (Hart et al., 2008; Masten, 2002). The different ‘components’ of the Framework, such as ‘Belonging’, ‘Learning’, ‘Coping’, and Knowledge of ‘core self’, are considered crucial in resilience-building (Southwick and Charney, 2013; Richardson, 2002; Robertson, 2016; Wiig et al., 2020) and were all apparent in the study findings. Belonging, as a component of resilience, was associated in the findings with friends, family, peers, and other internal and external support structures. Some participants also cited ‘belonging’ to the nursing profession as key to protecting their resilience; this is apparent in other work on resilience (Hart et al., 2012; Williamson et al., 2013). The importance of ‘career resilience’ and having a long-term career plan is cited as an enabler of resilience in previous studies exploring resilience with nursing students (Waddell et al., 2015).

The challenges to resilience highlighted by participants show the extent to which the healthcare system was unprepared for the pandemic. This led to ongoing uncertainty about the long-term repercussions for nursing students who were exposed to traumatic experiences on an ongoing basis and how their resilience might be impacted. The ways in which trauma is interpreted and the effects of individual trauma are dependent on the cultural lens through which the trauma is viewed (Buse et al., 2013). For students and staff alike, the traumas experienced through their nursing lenses were often related to illness and dying, which could be hard to process and adapt to. Throughout student nurse education, exposure to new experiences and witnessing challenging situations is a normal part of the professional development experience, enabling growth, reflection, and learning (Felstead et al., 2016). In these circumstances, students can normally draw on role models in the form of clinical and academic supervisors, who can provide reassurance through their own prior experiences (Felstead, 2013; Felstead; 2016); this aligns with approaches to Belonging and Learning in the Resilience Framework (Fig. 1). However, during the pandemic this embedded support was sometimes reported as missing, due to clinical and academic supervisors also experiencing uncertainty, loss, disruption, and other unprecedented challenges (Davey et al., 2022). This lack of reassurance and reliability from trusted role models is likely to have impacted nursing students’ resilience, with potentially far-ranging repercussions in the short and long-term.

During the pandemic nursing students’ desires to care for and support patients and their sense of pride in their vocation were often in conflict with concerns and anxieties around virus transmission. The increased sense of vulnerability experienced by nursing students, in terms of their own risk of catching the virus and passing it on to patients, is something that nursing students do not regularly experience on such a large scale. Whilst nurses are well- versed in infection risk and minimisation techniques as part of their clinical roles (Hinkin et al., 2014), the widespread and prolonged prevalence of the COVID-19 virus, both inside and outside the work environment, meant that students could never escape from this heightened sense of risk. These findings mirror a survey exploring qualified nurses’ experiences regarding risk to self and others in the pandemic and their concerns regarding lack of access to personal protective equipment and preparedness for redeployment (ICON, 2020). The Nursing and Midwifery Council states that part of nurses’ roles in the 21st century is to provide, lead, and coordinate compassionate, evidence-based, and person-centred care (NMC, 2018). If nursing students are concerned that by seeking to fulfil their nursing roles they are inadvertently putting patients at increased risk, this may undermine the essence of what it is to be a nurse, leading to ambiguity in terms of role identity, which can lead to increased role stress (Sun et al., 2016). For students, this role stress can be especially pertinent and long-lasting due to early clinical experiences being more formative in shaping their learning experiences and impacting their coping mechanisms, resilience, and future employment choices (Horsfall et al., 2012).

The sense of loss of control, uncertainty, and isolation experienced by many nursing students due to rapid and often poorly communicated changes to course programmes was often compounded by internal pressures to continue with their courses to support patients. This internal conflict between the personal and the professional can become testing in terms of resilience (Davey et al., 2022), in line with the Learning, Belonging, Coping, and Core Self components of the Resilience Framework (Fig. 1). For some, it can lead to a sense of increased empowerment and autonomy, fostering a sense of professional camaraderie and teamwork, whilst for others it can lead to burnout, with widespread repercussions on mental health (Henshall et al., 2020)**.**

A commonly described impact on nursing students related to their feeling the absence or minimisation of peer support networks due to increases in remote working, social isolation, and distancing requirements. Social isolation is known to have detrimental effects on well-being, with negative impacts on both physical and mental health (Kim et al., 2020). For nursing students, ongoing and relentless pressures experienced during clinical placements, at university and home, coupled with a lack of peer support, have the potential to test resilience to the breaking point. Accessing peer support has been recognised as a powerful coping mechanism (Cabilan and Kynoch, 2017). For nursing student peers, a shared understanding of what it feels like to be both an insider and an outsider during clinical placements may provide mutually beneficial support and increase a sense of belonging, especially during the upheaval of a pandemic.

The importance of social connections and networks was central to students acknowledging and working through issues and challenges, and their importance is supported in the wider literature (Reyes et al., 2015; Kim et al., 2020; Goldsmith et al., 2011). The essential mechanism of staying connected with others is cited in all evidence-based resilience theories as a protective factor against stress and burnout by building a sense of community (Southwick and Charney, 2013; Robertson, 2016; Richardson, 2002; Porteous and Machin, 2018; Wiig et al., 2020). This aligns with the resilience definition cited earlier, which states that nursing student resilience occurs through employing personal protective factors that help navigate stress and lead to enhanced coping abilities (Stephens, 2013). Supportive one-to-one relationships with peers, mentors, and supervisors were all cited by students as being of far greater value than the more formal support structures offered by the university or clinical settings, which were often poorly accessed. This highlights the importance of one-to-one, regular contacts with peers and course providers to leverage resilience and increase students’ ability to continue with their nursing programmes (Health Education England, 2018). The value of these one-to-one relationships in providing emotional and developmental support, as well as enabling problem solving, shared experiences, and the facilitation of tailored support, highlights the power of simple but effective communication channels in reaffirming self-efficacy, which is linked to enhanced resilience (Schwarzer et al., 2013). Despite the many technological and formal support networks and interventions available to students, we suggest that personalised one-to-one support was crucial for enabling students to feel valued. Communicating in this way can allow people to reflect on and reaffirm their contribution to and value within systems (Robbins et al., 2020); for students, this may allow them to recognise their roles in providing patient-centred care, positively reinforcing their role within the nursing profession.

Central to the Belonging component of the Resilience Framework is the formation of healthy relationships or communities. In our study, the importance of the workplace as a ‘community’ was highlighted during the pandemic, due to a heightened sense of camaraderie and belonging for some, which increased these nursing students’ sense of professional identity. This may impact on students’ personal sense of resilience by reiterating the value of the nursing profession and their roles within it; conversely, it may lead to feeling overwhelmed due to heightened pressures and feelings of guilt at not coping as well as their peers. This highlights the importance of supportive role-modelling from qualified nurses, as the desire to ‘fit in’, to be accepted as a team member, and to contribute to healthcare delivery is an important motivating factor for nursing students and can lead to the promotion of resilience (Felstead, 2013; Felstead et al., 2016).

This study had strengths and limitations. To our knowledge, it is the only qualitative study to have explored the needs of nursing students throughout the COVID-19 pandemic across the four nations of the UK. Utilising a four-nation approach enabled widespread representation across a range of geographical locations, universities, placement settings, and programme branches, including adult, child, mental health, and learning disabilities nursing. However, the recruitment strategy led to participant self-selection and may not be representative of all nursing students, providing a study limitation. For example, a disproportionate number of White students participated, potentially influencing the representativeness of the findings, and the number of male student nurses who participated was low. In addition, despite the study recruiting across the four UK nations, 71% of participants were recruited from England. Another study limitation is our large dataset enabled comprehensive accounts from a broad range of nursing students to be attained but was also challenging in terms of navigating the dataset and succinctly capturing the perspectives of all participants.

The study has generated insights that can be applied to nursing research, education, policy, and practice. In terms of research, future work could focus on developing and evaluating clear, structured communication support pathways for students that are tailored to their needs and preferences and can be adapted to deal with fast-changing contexts and healthcare landscapes to better protect future nurse trainees in other healthcare crises. This study has shown the value of personalised, tailored communication networks alongside more formal supportive interventions. Consideration of how this relational support is consistently provided within an overstretched and increasingly remote working system is key. Additionally, whilst many resilience enhancement interventions have been designed for healthcare staff (Kunzler et al., 2020; Foster et al., 2018; Slatyer et al., 2017; Henshall et al., 2020), few have focused on nursing students. As students are the future of the profession, more needs to be done to understand how they cope with highly-pressured situations to ensure they are well supported from the outset of their nursing careers. Consideration of how nurse education can support students to understand, prioritise, and maintain resilience in the workplace is also required. From a broader policy perspective, understanding how we can best mobilise student contributions to support systems during pandemics and other times of unpredictable service pressures is key, particularly whilst ensuring that their physical and emotional well-being needs are met through accessing relevant support networks.

## 7 Conclusions

In this study, we have identified the wide-ranging impact that the COVID-19 pandemic had on nursing students and their subsequent ability to remain resilient in an ever-changing and unstable environment. The immense value of communication and support networks from peers and placement providers has emerged as key to navigating the uncertainties that arose during this time. In addition, the extent to which students were able to navigate their personal and professional roles and identities played a role in their ability to cope with and continue along their training pathways. When the next public health crisis occurs, pre-registration nursing programmes should use our findings to inform their oversight by incorporating the variety of mechanisms that nursing students employ to maintain their resilience. Higher education institutions have a crucial role in ensuring that their links with healthcare organisations remain well connected and versatile, so that students can be fully supported and take advantage of enhanced communication mechanisms, mentorship and supervision, and flexible teaching and learning environments. Policy makers and educators should use the study findings to inform nursing education and practice as a way of reducing nursing student attrition and ensuring that current and future healthcare challenges are informed by the lessons from the COVID-19 pandemic that will support future nurse leaders.

## Funding sources

This study was funded by UKRI: ESRC via the grant call ‘Ideas to Address COVID-19′ (Grant Ref ES/VO158993/1). The funder was not involved in the study design; in the collection, analysis and interpretation of data; in the writing of the report; or in the decision to submit the article for publication.

## Acknowledgements

The authors would like to thank our participants for their contribution to this study. XX is supported by the National Institute for Health Research Oxford Health Clinical Research Facility. The views expressed are those of the authors and not necessarily those of the NIHR, UK National Health Service, or the UK Department of Health and Social Care.

## CRediT authorship contribution statement

**Catherine Henshall:** Conceptualization, Funding acquisition, Investigation, Methodology, Validation, Visualization, Writing – original draft, Writing – review & editing. **Zoe Davey:** Conceptualization, Data curation, Formal analysis, Funding acquisition, Investigation, Methodology, Project administration, Validation, Visualization, Writing – original draft, Writing – review & editing. **Clair Merriman:** Funding acquisition, Data curation, Formal analysis, Validation, Visualization, Writing – original draft, Writing – review & editing. **Laura Strumidlo:** Funding acquisition, Data curation, Formal analysis, Validation, Visualization, Writing – original draft, Writing – review & editing. **Laura Serrant:** Funding acquisition, Data curation, Formal analysis, Validation, Visualization, Writing – original draft, Writing – review & editing. **Jo Brett:** Conceptualization, Data curation, Formal analysis, Funding acquisition, Investigation, Methodology, Project administration, Supervision, Validation, Visualization, Writing – original draft, Writing – review & editing. **Eila Watson:** Conceptualization, Funding acquisition, Investigation, Methodology, Validation, Visualization, Writing – review & editing. **Jane V. Appleton:** Conceptualization, Funding acquisition, Investigation, Methodology, Validation, Visualization, Writing – review & editing. **Mary Malone:** Conceptualization, Data curation, Formal analysis, Funding acquisition, Investigation, Methodology, Project administration, Resources, Supervision, Validation, Visualization, Writing – review & editing. **Carrie Bradbury-Jones:** Funding acquisition, Validation, Visualization, Writing – review & editing. **Sarah Bekaert:** Data curation, Investigation, Project administration, Writing – review & editing. **Dan Butcher:** Data curation, Formal analysis, Funding acquisition, Validation, Visualization, Writing – review & editing. **Paul Dawson:** Data curation, Investigation, Project administration, Writing – review & editing. **Daniel Kelly:** Funding acquisition, Validation, Visualization, Writing – review & editing. **Sonja Mcllfatrick:** Funding acquisition, Validation, Visualization, Writing – review & editing. **Kinga Papiez:** Data curation, Investigation, Project administration, Writing – review & editing. **Anne Marie Rafferty:** Funding acquisition, Validation, Visualization, Writing – review & editing. **Pras Ramluggun:** Data curation, Investigation, Project administration, Writing – review & editing. **Mike Ramsay:** Funding acquisition, Validation, Visualization, Writing – review & editing. **Lynn Sayer:** Funding acquisition, Validation, Visualization, Writing – review & editing. **Marion Waite:** Data curation, Investigation, Project administration, Writing – review & editing. **Tessa Watts:** Funding acquisition, Validation, Visualization, Writing – review & editing. **Cate Wood:** Data curation, Investigation, Project administration, Writing – review & editing.

## Declaration of Competing Interest

None.

## Data Availability

Data will be made available upon reasonable request.

## Appendix Supplementary materials

Image, application 1

## References

[bib0001] Aiken L., Slone D., Brunynell S., Van den Heede K., Griffiths P., Busee R., Diomidous M., Kinnunen J., Koxka M., Lesaffre E., McHugh M., Moreno-Casbas M., Rafferty A., Schwendimann R., Scott A., Tishelman C., Achterberg T., Sermeus W. (2014). Nurse staffing and education and hospital mortality in nine European countries: a retrospective observational study. Lancet North Am. Ed..

[bib0002] American Association of Colleges of Nursing (AACN) (2022). AACN Press Release – Enrollment Increase. Available at: https://njccn.org/2022/04/08/aacn-press-release-enrollment-increase/Accessed 06.07.23.

[bib0003] Bagnasco A., Catania G., Gallagher A., Morley G. (2020). Media representations of nurses in the pandemic: just doing our job?. Nurs. Ethics.

[bib0004] Buse N., Bernacchio C., Burker E. (2013). Cultural variation in resilience as a response to traumatic experience. J. Rehabilit..

[bib0005] Cabilan C., Kynoch K. (2017). Experiences of and support for nurses as second victims of adverse nursing errors: a qualitative systematic review. JBI Datab. Syst. Rev. Implem. Rep..

[bib0006] Charles, A., and Ewbank, L. (2021). The Road to Renewal: five Priorities for Health and Care; King's Fund: London, UK. Available online: https://www.kingsfund.org.uk/publications/covid-19-road-renewal-health-and-care (accessed on 12 July 2022).

[bib0008] Davey Z., Jackson D., Henshall C. (2020). The value of nurse mentoring relationships: lessons learnt from a work-based resilience enhancement programme for nurses working in the forensic setting. Int. J. Ment. Health Nurs..

[bib0007] Davey Z., Srikesavan C., Cipriani A., Henshall C. (2022). It’s what we do: experiences of UK Nurses Working during the COVID-19 pandemic: impact on practice, identity and resilience. Healthcare.

[bib0010] Deogon G.S., Robbins T., Randeva M.S., Kyrou I., Sankar S., Randeva H.S., Murthy N. (2020). Managing high-acuity outpatient services during the COVID-19 pandemic: lessons from the acute diabetes foot service. Future Healthc. J..

[bib0011] Eick S., Williamson G., Heath V. (2012). A systematic review of placement-related attrition in nurse education. Int. J. Nurs. Stud..

[bib0012] Felstead I. (2013). Role modelling and students' professional development. Br. J. Nurs..

[bib0013] Felstead I., Springett K. (2016). An exploration of role model influence on adult nursing students’ professional development: a phenomenological research study. Nurse Educ. Today;.

[bib0014] Ferry A., Wereski R., Strachan F., Mills N. (2021). Predictors of UK healthcare worker burnout during the COVID-19 pandemic. QJM Int. J. Med..

[bib0015] Foster K., Shochet I., Wurfl A., Roche M., Maybery D., Shakespeare-Finch J., Furness T. (2018). On PAR: a feasibility study of the Promoting Adult Resilience programme with mental health nurses. Int. J. Ment. Health Nurs..

[bib0016] Gale N., Heath G., Cameron E., Rashid S., Redwood S (2013). Using the Framework Method for the Analysis of Qualitative Data in Multi-Disciplinary Health Research. BMC Med. Res. Methodol..

[bib0017] Goldsmith D., Albrecht T. (2011). The Routledge Handbook of Health Communication.

[bib0018] Hart A., Blincow D., Thomas H (2008). Resilient therapy: strategic therapeutic engagement with children in Crisis. Child Care Pract..

[bib0019] Hart, A., Blincow, D., and Cameron, J. (2012) *Resilience Framework (Adults)* Available at: http://www.boingboing.org.uk/index.php/resources/category/9-resilience-frameworks(Accessed 14 October 2019).

[bib0020] Hart A., Gagnon E., Eryigit-Madzwamuse S., Cameron J., Aranda K., Rathbone A., Heaver B. (2016). Uniting resilience research and practice with an inequalities approach. Sage Open.

[bib0021] Health Education England (2018).

[bib0023] Henshall C., Davey Z., Jackson D. (2020). The implementation and evaluation of a resilience enhancement programme for nurses working in the forensic setting. Int. J. Ment. Health Nurs..

[bib0024] Hinkin J., Cutter J. (2014). How do university education and clinical experience influence pre-registration nursing students' infection control practice? A descriptive, cross sectional survey. Nurse Educ. Today;.

[bib0025] Horsfall J., Cleary M., Hunt G. (2012). Developing a pedagogy for nursing teaching–learning. Nurse Educ. Today.

[bib0026] ICON, (2020) Survey of UK nurses and midwives' highlights their concerns about health, training and workload during COVID-19 (kcl.ac.uk).

[bib0027] Jackson D., Firtko A., Edenborough M. (2007). Personal resilience as a strategy for surviving and thriving in the face of workplace adversity: a literature review. J. Adv. Nurs..

[bib0028] Jones-Berry, S. (2021). Quitting before they qualify: what's behind the spike in nursing students dropping out? Available at: https://rcni.com/nursing-standard/newsroom/analysis/quitting-they-qualify-whats-behind-spike-nursing-students-dropping-out-177551. Accessed on: 02.09.2022.

[bib0029] Kim, U., Bhullar, N. and Jackson, D., (2020). Life in the pandemic: social isolation and mental health. https://onlinelibrary.wiley.com/doi/pdfdirect/10.1111/jocn. 15290. 10.1111/jocn.15290 32250493

[bib0030] Kunzler A.M., Helmreich I., Chmitorz A., König J., Binder H., Wessa M., Lieb K. (2020). Psychological interventions to foster resilience in healthcare professionals. Cochrane Datab. Syst. Rev..

[bib0031] Mahase E. (2022). Nurses vote to strike over pay, as other health workers are balloted. Br. Med. J..

[bib0033] McDonald G., Jackson D., Wilkes L., Vickers M. (2012). A work-based educational intervention to support the development of personal resilience in nurses and midwives. Nurse Educ. Today.

[bib0034] McGowan J.E., Murray K. (2016). Exploring resilience in nursing and midwifery students: a literature review. J. Adv. Nurs..

[bib0035] Morgantini L., Naha U., Wang H., Francavilla S., Acar Ö., Flores J., Crivellaro S., Moreira D., Abern M., Eklund M., Vigneswaran H., Weine S. (2020). Factors contributing to healthcare professional burnout during the COVID-19 pandemic: a rapid turnaround global survey. PLoS One.

[bib0036] Nurses.co.uk, (2021). Does The Increase In The Numbers Of Student Nurses This Year Augur Well for the Future? Available at: https://www.nurses.co.uk/blog/does-the-increase-in-the-numbers-of-student-nurses-this-year-augur-well-for-the-future/#:∼:text=In%20England%2C%2020%2C930%20students%20successfully,2020%2C%20to%201%2C290%20in%202021. Accessed on: 02.09.2022.

[bib0037] Nursing and Midwifery Council (2018).

[bib0039] Nursing and Midwifery Council (2020).

[bib0040] Porteous D.J., Machin A. (2018). The lived experience of first year undergraduate student nurses: a hermeneutic phenomenological study. Nurse Educ. Today.

[bib62] Reyes A., Andrusyszyn M-A., Iwasiw C., Forchuk C., Babenko-Mould Y. (2015). Nursing students’ understanding and enactment of resilience: a grounded theory study. J. Adv. Nurs..

[bib0041] Richardson G.E. (2002). The metatheory of resilience and resiliency'. J. Clin. Psychol..

[bib0042] Robertson I. (2016).

[bib0043] Robbins B., Davidhizar R. (2020). Transformational leadership in health care today. Health Care Manag. (Frederick).

[bib0044] Schwarzer R., Warner L. (2013). Resilience in Children, Adolescents, and Adults.

[bib0045] Shembavnekar, N., Woodham, E. (2021). What Might COVID-19 Mean for England's Nurse Supply? The Health Foundation: London, UK. Available online: https://www.health.org.uk/news-and-comment/blogs/what-might-covid-19-mean-for-englands-nursesupply (accessed on 12 July 2022).

[bib0046] Slatyer S., Craigie M., Heritage B., Davis S., Rees C. (2017). Evaluating the effectiveness of a brief mindful self-care and resiliency (MSCR) intervention for nurses: a controlled trial. Mindfulness;.

[bib0047] Southwick S.M., Charney D.S. (2013).

[bib0048] Stephens T. (2013). Nursing student resilience: a concept clarification. Nurs. Forum.

[bib0049] Sun L., Gao Y., Yang J., Zang X., Want Y. (2016). The impact of professional identity on role stress in nursing students: a cross-sectional study. Int. J. Nurs. Stud..

[bib0050] ten Hoeve Y., Castelein S., Jansen G., Roodbol P. (2017). Dreams and disappointments regarding nursing: student nurses' reasons for attrition and retention. A qualitative study design. Nurse Educ. Today.

[bib63] Traynor M. (2017). Critical Resilience for Nurses: An Evidence-based Guide to Survival and Change in the Modern NHS.

[bib0053] University and Colleges Admissions Service (UCAS), (2021). Nursing Applications Soar as UCAS Publishes Latest Undergraduate Applicant Analysis. Available at: https://www.ucas.com/corporate/news-and-key-documents/news/nursing-applications-soar-ucas-publishes-latest-undergraduate-applicant-analysis#:∼:text=Total%20applications%20for%20nursing%20courses,39%25%20rise)%20have%20applied. Accessed on 02.09.2022.

[bib0054] Vindrola-Padros C., Andrews L., Dowrick A., Djellouli D., Fillmore H., Bautista Gonzalez E., Javadi D., Lewis-Jackson S., Manby L., Mitchinson L., Mulcahy Symmons S., Martin S., Regnold N., Robinson H., Sumray K., Singleton G., Syversen A., Johnson G. (2020). Perceptions and experiences of healthcare workers during the COVID-19 pandemic in the UK. Brit. Med. J. Open.

[bib0055] Waddell J., Spalding K., Canizares G., Navarro J., Connell M., Jancar S., Stinson J., Victor C. (2015). Integrating a career planning and development program into the baccalaureate nursing curriculum: part I. Impact on students' career resilience. Int. J. Nurs. Educ. Scholarsh..

[bib0056] Wiig S., Aase K., Billett S. (2020). Defining the boundaries and operational concepts of resilience in the resilience in healthcare research program. BMC Health Serv. Res..

[bib0057] Willan J., King A.J., Jeffery K., Bienz N. (2020). Challenges for NHS hospitals during COVID-19 epidemic. Br. Med. J..

[bib0058] Williamson G.R., Health V., Proctor-Childs T. (2013). Vocation, friendship and resileince: a study exploring nursing student and staff views on retention and attrition. Open Nurs J.

[bib0059] Wilson, G. (2022). Why nursing students are dropping out. The Junction. Available at: https://junctionjournalism.com/2022/05/18/why-nursing-students-are-dropping-out/ Accessed on: 02.09.2022.

[bib0060] World Health Organisation (2009).

[bib0061] Zhou M., Kan M.Y. (2021). The varying impacts of COVID-19 and its related measures in the UK: a year in review. PLoS One.

