# Supplementary material for: The impact of the COVID-19 pandemic on nursing students’ navigation of their nursing programmes and experiences of resilience. A qualitative study
Source: Int J Nurs Stud Adv. 2023 Jul 28;5:100146. doi: 10.1016/j.ijnsa.2023.100146 (PMC11080317; doi:10.1016/j.ijnsa.2023.100146)
Supplement: Supplementary file 1 [file mmc1.docx]

**Appendix 1 – Pre-placement Survey**

**About your placement:**

Please confirm the start and end dates of the placement you are going to be on while you participate in this study:

Start date:

End date:

What type of placement is this?

- Supernumerary placement
- Opt-in paid placement

**About you:**

What is your age (in years)?

What is your marital status?

- Married/defacto/civil partnership
- Divorced or separated
- Widowed
- Single
- In a relationship
- Other (please specify):

Do you have any children?

- Yes (how many? what ages?)
- No

Do you have any other dependents?

- Yes (please specify):
- No

**Your feelings about your placement:**

What were your feelings ahead of starting this placement during the COVID-19 pandemic? (Please select all that apply)

- Excited
- Apprehensive
- Motivated
- Nervous
- Frightened
- Determined
- Other (please specify):

How well-prepared did you feel starting out on the placement?

- Very well prepared
- Well prepared
- Somewhat prepared
- Not very prepared
- Not at all prepared
